# Neoadjuvant therapy is associated with lower margin positivity rates after Pancreaticoduodenectomy in T1 and T2 pancreatic head cancers: An analysis of the National Cancer Database

**DOI:** 10.1016/j.sopen.2020.12.001

**Published:** 2020-12-16

**Authors:** Stephanie H. Greco, David A. August, Mihir M. Shah, Chunxia Chen, Dirk F. Moore, Monika Masanam, Amber L. Turner, Salma K. Jabbour, Parisa Javidian, Miral S. Grandhi, Timothy J. Kennedy, H. Richard Alexander, Darren R. Carpizo, Russell C. Langan

**Affiliations:** a Gastrointestinal and Hepatobiliary Oncology, Rutgers Cancer Institute of New, Jersey; b Department of Surgery, Rutgers Robert Wood Johnson University Medical School; c Department of Surgery, RWJBarnabas Health, Saint Barnabas Medical Center; d Division of Surgical Oncology, Department of Surgery, Emory University; e Biostatistics, Rutgers Cancer Institute of New, Jersey; f Division of Radiation Oncology, Rutgers Cancer Institute of New, Jersey; g Department of Pathology, Rutgers Robert Wood Johnson University Hospital

## Abstract

**Background:**

Neoadjuvant therapy (NAT) for T1/T2 pancreatic adenocarcinoma (PDAC) prior to pancreaticoduodenectomy remains controversial. We compared positive margin rates in patients with clinical T1&T2 tumors who did and did not receive NAT.

**Methods:**

The National Cancer Database (NCDB) found clinical T1&T2 PDAC patients who underwent pancreaticoduodenectomy from 2004 to 2014. Univariate and multivariate regression determined factors associated with a positive margin and survival.

**Results:**

9795 patients underwent surgery for clinical T1 or T2 pancreatic head adenocarcinoma. 8472 patients had data regarding use of neoadjuvant and adjuvant therapies; of which, 774 (9.1%) received NAT and 435 (5.1%) received both chemotherapy and radiation therapy. NAT was found to lower positive margin rates from 21.8 to 15.5% (p < 0.0001) and when radiation was added this rate dropped to 13.4%. Positive margins were associated with worse overall survival (14.9 vs. 23.9 months; HR 1.702, *p* < 0.0001).

**Conclusions:**

NAT is associated with a reduced positive margin rate in patients with T1 and T2 tumors. These findings support ongoing and future clinical trials of NAT in T1 and T2, early stage PDAC to determine impacts on survival.

## Highlights

• Neoadjuvant therapy for resectable pancreas cancer is controversial
• Neoadjuvant therapy can improve margin positivity
• Margin positivity is associated with inferior survival
• Even small, resectable pancreas cancers may benefit from neoadjuvant therapy

## Introduction

Pancreatic ductal adenocarcinoma (PDAC) is the third leading cause of cancer death in the United States and carries a poor prognosis with an overall five-year survival rate of less than ten percent [1]. Surgery remains the only potentially curative treatment. However, the overwhelming majority of patients, even those undergoing surgical resection with curative intent, experience recurrences, highlighting the importance of adjuvant therapies. Post-operative systemic chemotherapy has been shown to be effective in randomized clinical trials [2]. The role of radiation in the postoperative setting, however, remains controversial [[2], [3], [4]].

To date, many factors have been identified as predictors of outcomes following resection of PDAC. These include tumor size, location, histologic grade, nodal status, and surgical margin status [5]. In particular, positive margin rate has garnered more attention in recent years because of its association with survival outcomes. In general, the rate of positive margin after pancreaticoduodenectomy for PDAC is high, reaching 25% even in patients with disease evaluated as resectable using modern imaging techniques; the positive margin rate is even higher in patients with borderline resectable disease [6,7]. In fact, due to the high positive margin rate in borderline resectable patients, neoadjuvant chemotherapy and/or radiation (NAT), is emerging as the standard of care [8,9]. This paradigm shift occurred following studies which suggested that patients with borderline-resectable PDAC who received NAT were less likely to have positive surgical margins and less likely to have positive lymph nodes [10,11]. In addition, patients who received preoperative radiation therapy were shown to have a lower rate of positive margins and negative lymph nodes on surgical pathology as compared to those who received postoperative radiation [12].

Even though margin positivity rates remain high in patients staged as resectable, NAT remains controversial for this patient population. Therefore, we sought to evaluate the impact of NAT on positive margin rates after pancreaticoduodenectomy in the most favorable PDAC population. As a surrogate for patients with resectable disease likely to be treated with surgery alone or surgery and adjuvant therapy only, we used the National Cancer Database (NCDB) to identify those patients with clinical T1 and T2 tumors, and analyzed the positive margin rate and overall survival in these subjects. We hypothesized that surgical resection for T1 and T2 PDAC would be associated with a high rate of positive margins and that patients undergoing NAT would have lower rates of positive margins.

## Materials and methods

### Data Source

We conducted a retrospective review of patients undergoing definitive surgery for clinical T1 or T2 pancreatic head adenocarcinoma using the National Cancer Database (NCDB) from 2004 to 2014. The NCDB is a joint project of the Commission on Cancer of the American College of Surgeons and the American Cancer Society. The NCDB, established in 1989, is a nationwide, facility-based, comprehensive clinical surveillance resource oncology data set that currently captures 70% of all newly diagnosed malignancies in the US annually. Data was extracted from a de-identified file provided by the NCDB. The Saint Barnabas Medical Center, RWJBarnabas Health Institutional Review Board granted this study an expedited status.

We used 22 of 58 histology codes provided by the NCDB to select patients with PDAC based on the International Classification of Diseases for Oncology, 3rd edition (ICD-0-3.1) and consultation with our gastrointestinal pathologist (Appendix 1). Since the NCDB does not have a variable which distinguishes between resectable and borderline resectable disease, we chose to use clinical T1 and T2 status as a surrogate for tumors likely to have been operated on with the presumption that negative surgical margins would result. Thus, only patients with clinical T1 or T2 pancreatic head cancer (T1 = limited to the pancreas and < 2 cm; T2 = limited to pancreas and greater than 2 cm) and a single malignant primary were included in the analysis. Staging was based on the AJCC 6th and 7th editions (same T staging) based on the timeframe of this data collection. Patients were then subcategorized into treatment groups according to the use of 1) neoadjuvant therapy: defined as either preoperative chemotherapy and/or radiation, or 2) no neoadjuvant therapy. Twenty-one patients received neoadjuvant radiation only and were excluded from the final analysis. Clinical and pathologic variables were then extracted from the database. Our primary outcome was the rate of positive margin which is defined in the NCDB as either a gross or microscopically positive surgical margin. Therefore, we defined a R0 resection as any negative margin (0 mm or greater), which is consistent with NCCN reporting guidelines. R1 (microscopic residual tumor) and R2 (macroscopic residual tumor) margins were included together as a positive margin. Our secondary outcomes were overall survival, and 30-day and 90-day mortality.

### Statistical analysis

Continuous variables were reported using mean and standard deviation and analyzed using the Students t test. Categorical variables were reported using frequencies and proportions and evaluated using the Chi-square test. Overall survival was defined as the time from diagnosis to the date of last contact or death and was analyzed using the Kaplan–Meier method [13]. We subsequently repeated the survival analysis by excluding patients who did not survive 6 months from the day of diagnosis in order to eliminate bias since patients undergoing neoadjuvant chemotherapy and/or radiation would have already survived at least several months prior to surgery. This strategy is known as the “landmark” method and avoids the improper introduction of a time-dependent variable [14].

Multivariate analysis was performed using stepwise regression for clinical variables that were associated with margin positivity including: age, gender, race, year of diagnosis (before or after 2011), Charlson-Deyo morbidity score [15], facility type, facility location, clinical N stage, tumor size, and insurance status. The influence of the above variables as well as margin status, tumor grade, and pathologic N and T stage on OS was then determined by adjusting for the clinical variables identified. All statistical calculations were carried out using SAS software version 9.4 (SAS Institute, 2018).

## Results

### Clinical and Demographic Variables

**Fig. 1:**
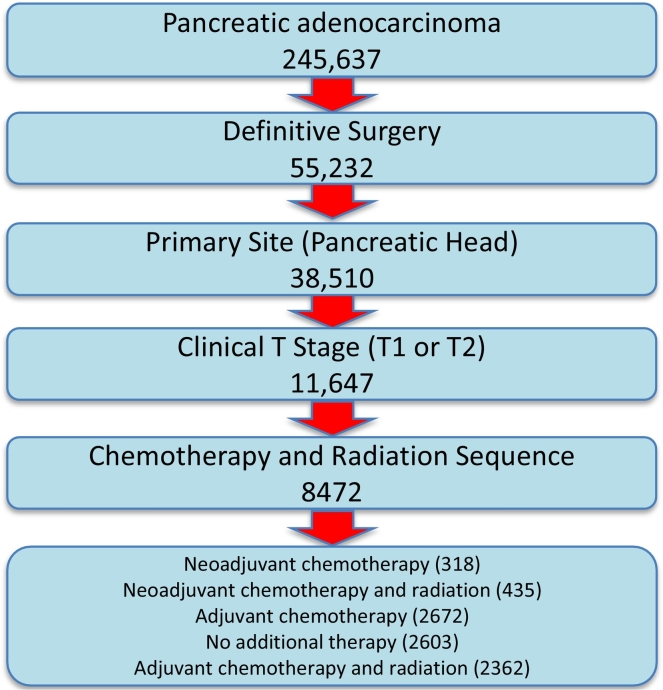
Consort diagram including study population inclusion criteria.

A total of 11,647 patients were identified who underwent curative-intent surgery for clinical T1 and T2 PDAC. 9795 of these patients had only a single malignant primary and 8472 had data for use of adjuvant therapies and were included in the final analysis (Fig. 1). Of the 774 patients who received NAT, 318/774 (41%) had neoadjuvant chemotherapy whereas 435/774 (56%) had neoadjuvant chemotherapy and radiation. Twenty-one patients had only neoadjuvant radiation only and were excluded from the final analysis, as this treatment is not considered standard of care. Based upon the remaining 758 patients, only 9% of patients received NAT followed by surgery which was consistent with our analysis that most patients with T1 and T2 tumors were in the resectable category. 2672/8472 patients (32%) received adjuvant chemotherapy alone, 2362/8472 (28%) received adjuvant chemotherapy and radiation, and 2603/8472 (31%) had no additional treatment other than surgery. 61/8472 (0.7%) patients received adjuvant radiation only.

Table 1 shows a comparison of baseline characteristics according to the use of NAT. Many clinical parameters were statistically different between the two groups (Table 1**)**. In particular, patients who received NAT were more likely to be slightly younger (average age 63.4 vs. 66.1 years, p < 0.0001), male (55.2% vs. 50.4%, p = 0.011), had only a single comorbidity (94.8% vs. 92.7%, p = 0.027), be treated at an Academic cancer center (60.1% vs. 54.0%, p = 0.001), have private insurance (48.6% vs. 38.0%, p < 0.0001) and have clinically node positive disease (26.5% vs. 17.6%, p < 0.0001). Also, the proportion of patients with T1c (1–2 cm) or T2 (2–4 cm) tumors was significantly higher in the NAT group (97.2% vs. 96.8%, p = 0.008). In addition, patients who received NAT were more likely to be diagnosed after 2011. Of note, NAT did not predict 30 or 90-day mortality, or tumor grade.

**Table 1:** Comparison of Baseline Characteristics According to Neoadjuvant Treatment.

	NAT (n = 774)	No NAT (n = 7698)	*p*-value
Age (mean)	63.4 ± 10.1	66.1 ± 10.4	< 0.0001
Gender			0.011
Male	55.2%	50.4%	
Female	44.8%	49.6%	
Race			0.029
Caucasian	85%	86.8%	
Black	3%	3.9%	
Other	12%	9.3%	
Charlson-Deyo Comorbidity Score			0.027
0–1	94.8%	92.7%	
> 2	5.2%	7.3%	
Facility Location			0.032
Northeast	19.9%	20.4%	
Midwest	30.3%	27.7%	
South	38.7%	37.1%	
West	11.1%	14.8%	
Facility Type			0.001
Academic	60.1%	54.0%	
Comprehensive Cancer Center	24.4%	30.6%	
Community	2.8%	3.7%	
Other	12.7%	11.7%	
Year of Diagnosis			< 0.001
Before 2011	32.2%	44.9%	
After 2011	67.8%	55.1%	
Median Income Quartile			0.054
1	15.7%	15.7%	
2	26.8%	23.3%	
3	27.6%	27.0%	
4	29.9%	34.0%	
Insurance Type			< 0.0001
Private	48.6%	38.0%	
Medicare/Medicaid	46.8%	57.7%	
Uninsured	2.6%	3.0%	
Other	2%	2.3%	
Tumor Size (mm)	32.9 ± 32	31.3 ± 18.6	0.185
Clinical T Stage (T1c or T2)	97.2%	96.8%	0.008
Clinical N Stage			< 0.0001
N0	73.5%	82.4%	
N1	26.5%	17.6%	

Key: NAT = neoadjuvant therapy.

### Positive Margin Rate

We found that the overall rate of a positive margin for all T1 and T2 tumors was 21.3%. The rate of positive margin was significantly lower in patients who received NAT versus patients who had no NAT (15.5% vs. 21.8%, *p* < 0.0001). The majority of positive margin resections in both the NAT and no NAT groups were R1 (microscopic residual tumor) at 57.0% and 43.3% respectively. Macroscopic (R2) residual tumor was present in only 3.1% and 1.2% of both groups, and residual tumor NOS or unknown margins were present in the remainder of patients. Patients who received neoadjuvant chemotherapy and radiation had the lowest positive margin rate overall, these patients had significantly lower positive margin rates compared to patients who received neoadjuvant chemotherapy only (13.4% vs. 18.6%, *p* < 0.001). Multivariate analysis using stepwise regression for clinical variables that were associated with positive margins identified several factors to be associated with a lower rate of positive margin after surgery (Table 2). These included: lower Charlson-Deyo comorbidity score of 0–1 (p = 0.009), treatment at an Academic facility (p = 0.016), clinical N0 stage (p = 0.012), smaller tumor size (p < 0.0001), and the use of NAT (p = 0.0004). Age, sex, race, year of diagnosis (before or after 2011) and facility location were not associated with margin status.

**Table 2:** Multivariate Analysis for Factors Predictive of a Positive Surgical Margin.

	OR (95% CI)	*p*-value
Charlson-Deyo Score (0–1)	0.742 (0.592–0.928)	0.009
Facility Treatment Type		0.016
Comprehensive Cancer Center	1.152 (1.001–1.327)	
Community	1.022 (0.718–1.456)	
Other	1.329 (1.100–1.607)	
Clinical N0	0.819 (0.700–9.57)	0.012
Tumor Size		< 0.0001
T1c	1.569 (0.746–3.302)	
T2	2.855 (1.375–5.929)	
NAT	0.645 (0.506–0.822)	0.0004

OR = Odds ratio; NAT = neoadjuvant therapy.

For Charlson-Deyo Score, above 1 is the reference.

For Facility Type, Academic Facility is the reference.

For Clinical Stage, N1 is the reference.

For Tumor Size, T1a is the reference.

For NAT, no NAT is the reference.

### Survival

**Fig. 2:**
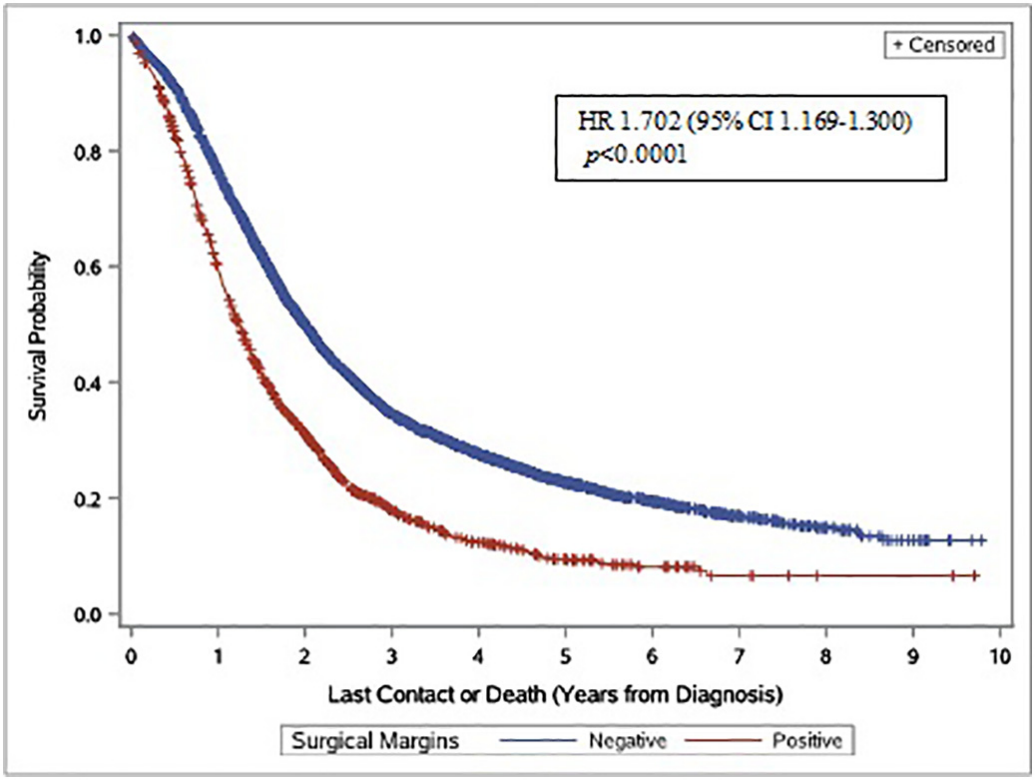
Margins are Predictive of Overall Survival (Kaplan–Meier curve for overall survival according to surgical margin status. Key: HR = Hazard ratio).

**Fig. 3:**
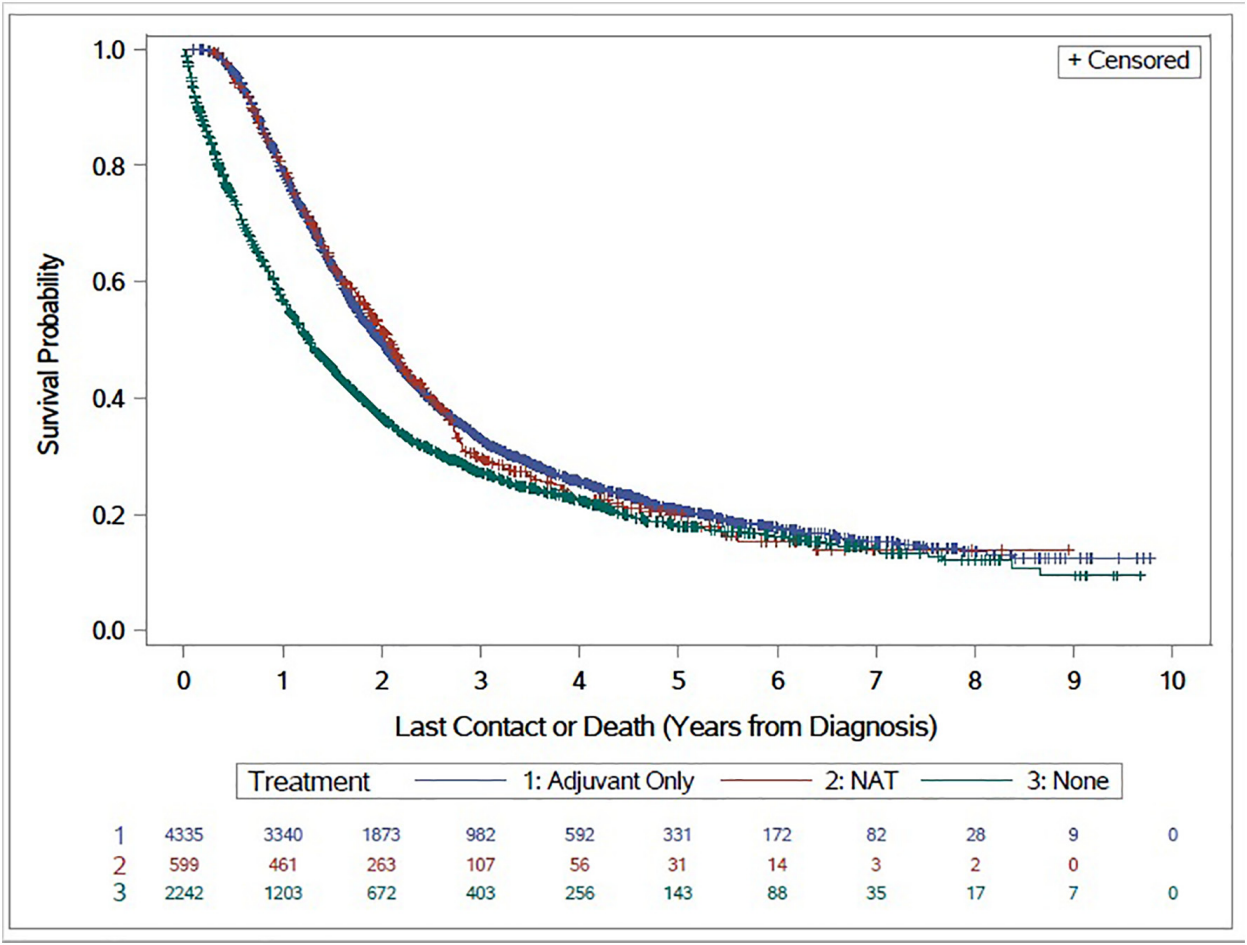
NAT or Adjuvant Therapy Improves Overall Survival (Kaplan–Meier curve for overall survival according to the type of therapy received. Key: NAT = neoadjuvant therapy; HR = Hazard ratio).

**Fig. 4:**
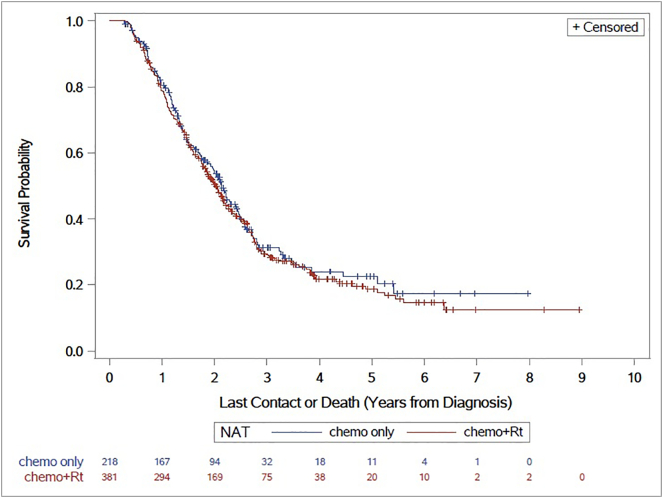
Overall Survival is not affected by Type of NAT (Kaplan–Meier curve for overall survival according to the type of neoadjuvant therapy received. Key: NAT = neoadjuvant therapy; HR = Hazard ratio; Rt = radiation).

Surgical margin status was associated with median overall survival, (14.9 months in patients not receiving NAT vs. 23.9 months in patients receiving NAT; HR 1.702, CI 1.169–1.300, *p* < 0.0001) (Fig. 2). Furthermore, use of NAT or adjuvant therapy was associated with an improved median OS (NAT = 24.7 months; HR 0.712, 95% CI 1.90–2.20; adjuvant therapy = 23.8 months, HR 0.706, 95% CI 1.90–2.05 versus 15.21 months in surgery only patients, *p* < 0.0001) (Fig. 3). Of note, we observed no survival difference between patients who received neoadjuvant chemotherapy and/or radiation versus those who only received neoadjuvant chemotherapy (24.3 vs. 25.6 months) (Fig. 4). Additionally, we found no difference in overall survival in patients who received NAT to those who received adjuvant therapy. When we excluded patients who died within 6 months from the day of diagnosis using the “landmark” method, all results were similar. This suggests that the absence of a benefit of NAT on overall survival is not due to a time-dependent confounder.

The following factors were independently associated with improved overall survival after multivariate analysis: younger age (*p* < 0.0001), Caucasian race (*p* *=* 0.048), lower Charlson-Deyo Score (0–1) (*p* *=* 0.020), treatment at an Academic facility (*p* *=* 0.004), treatment in the Northeast region (*p* *=* 0.015), clinical N0 stage (*p* *=* 0.007), pathologic N0 stage (*p* *<* 0.0001), smaller tumor size (*p* < 0.0001), lower grade (*p* < 0.0001), negative surgical margin (*p* < 0.0001), and NAT (*p* < 0.0001). Note, however, that although this regression analysis suggested that the use of NAT was a predictor of improved survival, in the Kaplan–Meier analysis presented above, NAT showed no advantage in OS in comparison to adjuvant therapy.

### Positive Lymph Node Rate

NAT was associated with lower rates of pathologically positive lymph nodes. Patients who received NAT had a 6% pathological N1 status versus 13.6% in patients who did not receive NAT (*p* *<* 0.0001). The mean number of positive regional lymph nodes was 1.44 versus 2.30 respectively between the two groups (*p* *<* 0.0001).

### 30 day and 90 day mortality

There was no difference in either 30-day or 90-day postoperative mortality between the NAT and no NAT groups (8.5% vs. 8.1% and 8.4% vs 10.8% respectively, p = 0.07; p = 0.8).

## Discussion

The current NCCN recommendation for patients with resectable or early-stage disease is surgery followed by adjuvant chemotherapy with or without radiation [16,17]. However, neoadjuvant therapy may be considered in select high-risk patients such as those with larger tumors, significantly elevated CA 19–9, or large regional lymph nodes. This approach has become the standard of care for patients with borderline resectable disease who are at higher risk of having a positive surgical margin. Despite lack of randomized data there is increasing utilization of neoadjuvant therapy for patients with resectable disease as well.

In this study, we have found that NAT is associated with a lower margin positive rate after resection in patients with small T1 and T2 tumors (15.49% vs. 21.84%) and even more so with the use of neoadjuvant combined chemotherapy and radiation therapy (13.40%). Additionally, margin rates and NAT were independent predictors of overall survival, although we found no differences in survival between NAT patients and those who received adjuvant therapy. Therefore, we present our data as hypothesis generating for more robust analyses.

A major theoretical advantage of the use of NAT is a potential to decrease tumor size and therefore decrease rates of positive margin. The overall rate of margin positive resection remains high at approximately 25–35% [18]. In our study, the rate of a positive margin for patients with T1 and T2 tumors, presumably the most favorable tumors, was still 21%. This is consistent with rates reported in the literature using the NCDB database [7,19]. This is noteworthy, since a positive margin has been associated with poorer overall survival [[20], [21], [22]]. Additionally, prior data suggests that margin distance (< 1.0 mm or < 1.5 mm and location (PV-SMV or SMA) are associated with a worse survival in specific subgroups [23,24]. Indeed, our analysis also demonstrates this association with a median OS of patients with a positive margin versus negative margin of 14.88 versus 24.88 months. Lastly, there is evidence that NAT may not increase the rate of postoperative complications and we found no differences in either 30-day or 90-day perioperative mortality between our two groups [25].

In contrast, several potential disadvantages of NAT have been suggested. One is that NAT can potentially add significant delay to surgery due to the inherent risks of treatment, thereby delaying potentially curative treatment in patients who have resectable disease. Moreover, some studies have found that upwards of 30% of patients who begin NAT do not make it to surgical resection due to complications [26,27]. However, a recent retrospective review of 1600 patients using the NCDB from 2006 to 2014 found an associated long-term survival benefit in patients with PDAC who underwent surgery > 12 weeks after completion of neoadjuvant chemoradiation [28]. A second criticism of NAT use is the lack of standardization. Currently, there is significant variability in the type of chemotherapy and whether or not radiation therapy is used as a component of therapy. Also, the use of NAT usually requires an upfront biopsy and potentially biliary stenting, both of which are not always needed to proceed with surgery, and carry risks of pancreatitis and other related complications.

Interestingly, we found no differences in survival between NAT patients and those who received adjuvant therapy. The lack of difference in overall survival between these two groups is likely due to several factors. First, there are many potential confounders such as the heterogeneity of potential NAT or adjuvant regimens, and the limitation of the NCDB to determine factors such as type of chemotherapy and duration of treatment. Additionally, we were unable to discern which patients received both NAT and adjuvant therapy or which patients completed the intended course of postoperative adjuvant treatment. Lastly, for patients with smaller tumors and early stage disease, our retrospective study may be unpowered to demonstrate a survival difference in this particular cohort. In fact, similar retrospective analyses have also failed to demonstrate improved overall survival in patients with clinical stage I disease who receive NAT versus adjuvant therapy [29].

Our findings are supported by several other retrospective analyses. For example, Mirkin et al. assessed the use of NAT in 18,332 patients also using the NCDB from 2003 to 2011 [29]. Furthermore, NAT was associated with improved OS versus surgery alone (median OS 24.84 versus 18.27 months, *p* < 0.0001), but not in patients with clinical stage I disease (median OS 24.94 months). This finding is thought-provoking and deserves further analyses. However, patients with clinical stage III disease who received NAT survived 8 months longer (22.57 vs. 14.55 months, *p* < 0.0001) versus those who received adjuvant therapy alone. These data suggest that perhaps only patients with T3 or greater tumors achieve benefit from NAT [7].

A similar study performed by Mokdad et al. using the NCDB from 2006 to 2012 including 15,237 with PDAC showed that patients who received resection first as compared to NAT were more likely to have a positive resection margin (24% vs. 17%) and positive lymph nodes (HR 1.68 (95% CI 1.56–1.82), *p* < 0.01); both of which were independent predictors of reduced overall survival (HR 1.59, (95% CI 1.48–1.71), *p* < 0.01). However, there was a minimal median overall survival benefit observed when comparing NAT to upfront resection with adjuvant therapy (26 vs. 23 months, *p* < 0.01) [6]. Additionally, Youngwirth et al. found that NAT was associated with improved OS versus no NAT in stage I or II PDAC following pancreaticoduodenectomy (median OS 24.3 vs. 18.7 months, *p* = 0.005) [19].

While discussing pancreatic margins we would be remiss not to include an analysis by Kooby et al. that reported the value of intraoperative pancreatic neck margin in 1399 patients [30]. This group found that additional resection to achieve a negative margin after a positive frozen section was not associated with improved OS. Interestingly and more recently, Zhang et al. found conflicting results. They concluded that negative resection margin based on frozen section was an independent predictor of OS [31]. We conclude that the question on whether margin status affects OS remains nebulous and warrants further analyses.

Limitations to our study include its retrospective design, small sample size, and the inherent selection bias with a national dataset. Specifically, our NAT cohort included only those who had NAT and made it to surgery, as it is not possible in the NCDB to determine which patients received neoadjuvant therapy and did not make it to surgery. Therefore, we are unable to do an intention-to-treat analysis. We are also unable to determine which patients received both neoadjuvant and adjuvant therapy, and this may have affected the results. In addition, we were unable to classify patients according to resectable versus borderline status based on the NCCN criteria and therefore we used clinical T1 and T2 tumors as a surrogate marker of resectable disease. Our findings are dependent upon accuracy of the staging registered in the dataset. Thirdly, this study is limited by the lack of data on the specific type and length of neoadjuvant and adjuvant therapies, and on progression during neoadjuvant therapy. Lastly, there is no data on local recurrence rates or disease-free survival.

Whether or not neoadjuvant therapy followed by surgery truly improves outcomes over surgery followed by adjuvant therapy is currently has been investigated in the recently published phase III PREOPANC trial of both resectable and borderline PDAC patients [32]. This study showed that the R0 resection rate was significantly higher in the preoperative gemcitabine-based chemoradiotherapy group (71% vs. 40%, p < 0.001), and was associated with better disease-free survival (HR 0.73 [0.55 to 0.96], p = 0.032) and locoregional failure-free interval (HR 0.56 [0.38 to 0.83], p-0.0034). The preoperative chemoradiotherapy group also had lower rates of pathologic lymph nodes, perineural, and venous invasion (p < 0.001, p < 0.001, p = 0.024 respectively). However, the median overall survival was not statistically significant between groups (16 vs. 14.3 months, HR 0.78 [0.53–1.05], p = 0.96). was improved for preoperative chemoradiotherapy patients who received had surgery and started adjuvant therapy (35.2 vs. 19.8 months, p = 0.029). Although, there was no difference in primary and secondary outcomes for resectable disease, 89% of patients completed preoperative treatment versus only 58% completed adjuvant therapy in both groups. This suggests better compliance with preoperative therapy. Furthermore, this trial used gemcitabine based chemotherapy, whereas FOLFIRINOX is a more recent popular regimen. Preoperative FOLFRINOX is currently being investigated in the PREOPANC 2 trial (NTR7292). Lastly, recently presented data from the phase II/III Prep-02/JSAP-05 trial showed a benefit of preoperative gemcitabine and S-1 over immediate surgery for resectable PDAC with a median OS of 36.7 versus 26.6 months [33]. Additional randomized trials for patients with resectable disease are ongoing using different neoadjuvant regimens including the NEOPA trial using gemcitabine with external beam radiation [34].

## Conclusion

Neoadjuvant chemotherapy and radiation is associated with lower rates of positive margin after pancreaticoduodenectomy for small (T1 and T2) PDAC. Given that the positive margin rate is high even with small T1 and T2 tumors, these results support further investigation of NAT in clinical trials of up front resectable patients with the aim of increasing overall survival.

## Conflict of interest

The authors have nothing to disclose.

## Funding sources

None.

## Author Contribution

Study Design: SG, DA, MS, SJ, PJ, MG, TK, HA, DC, RL; Data Acquisition: SG, CC, MM, AT; Methodology and Formal Analysis: DM; Data Interpretation: SG, DA, MS, SJ, PJ, MG, TK, HA, DC, RL; Administration: CC, MM, AT; Manuscript Drafting: SG, RL; Manuscript Editing: All authors critically reviewed and contributed to the revision of the manuscript All authors approved the final version of the manuscript for publication.

All authors critically reviewed and contributed to the revision of the manuscript All authors approved the final version of the manuscript for publication.

## Data Availability Statement

The data that support the findings of this study are available from the National Cancer Database. Restrictions apply to the availability of these data, which were used under license for this study. Data are available from the National Cancer Database if permission is granted from the National Cancer Database.

## Figures and Tables

**Table:** 

ICD-0-3.1 Code	Histology
8012	Large cell carcinoma, NOS
8021	Carcinoma, anaplastic NOS
8035	Carcinoma with osteoclast-like giant cells
8050	Papillary carcinoma, NOS
8140	Adenocarcinoma, NOS
8144	Adenocarcinoma, intestinal type
8211	Tubular adenocarcinoma
8255	Adenocarcinoma with mixed subtypes
8260	Papillary adenocarcinoma, NOS
8290	Oxyphilic adenocarcinoma
8310	Clear cell adenocarcinoma, NOS
8450	Papillary cystadenocarcinoma, NOS
8453	Intraductal papillary-mucinous carcinoma, invasive
8470	Mucinous cystadenocarcinoma, NOS
8471	Papillary mucinous cystadenocarcinoma
8480	Mucinous adenocarcinoma
8481	Mucin-producing adenocarcinoma
8490	Signet ring cell carcinoma
8500	Infiltrating duct carcinoma, NOS
8503	Intraductal papillary adenocarcinoma with invasion
8521	Infiltrating ductular carcinoma
8576	Hepatoid adenocarcinoma

NOS = not otherwise specified.
